# First two months of the 2019 Coronavirus Disease (COVID-19) epidemic in China: real-time surveillance and evaluation with a second derivative model

**DOI:** 10.1186/s41256-020-00137-4

**Published:** 2020-03-02

**Authors:** Xinguang Chen, Bin Yu

**Affiliations:** 1 Department of Epidemiology, University of Florida, 2004 Mowry Road, Gainesville, FL USA; 2 Global Health Institute, Wuhan University, Wuhan, Hubei Provinces China

**Keywords:** COVID-19, 2019-nCoV, outbreak, Second derivative, Infectious disease epidemic, Dynamic modeling

## Abstract

**Background:**

Similar to outbreaks of many other infectious diseases, success in controlling the novel 2019 coronavirus infection requires a timely and accurate monitoring of the epidemic, particularly during its early period with rather limited data while the need for information increases explosively.

**Methods:**

In this study, we used a second derivative model to characterize the coronavirus epidemic in China with cumulatively diagnosed cases during the first 2 months. The analysis was further enhanced by an exponential model with a close-population assumption. This model was built with the data and used to assess the detection rate during the study period, considering the differences between the true infections, detectable and detected cases.

**Results:**

Results from the second derivative modeling suggest the coronavirus epidemic as nonlinear and chaotic in nature. Although it emerged gradually, the epidemic was highly responsive to massive interventions initiated on January 21, 2020, as indicated by results from both second derivative and exponential modeling analyses. The epidemic started to decelerate immediately after the massive actions. The results derived from our analysis signaled the decline of the epidemic 14 days before it eventually occurred on February 4, 2020. Study findings further signaled an accelerated decline in the epidemic starting in 14 days on February 18, 2020.

**Conclusions:**

The coronavirus epidemic appeared to be nonlinear and chaotic, and was responsive to effective interventions. The methods used in this study can be applied in surveillance to inform and encourage the general public, public health professionals, clinicians and decision-makers to take coordinative and collaborative efforts to control the epidemic.

## Introduction

The epidemic of COVID-19 is caused by a novel virus first detected in Wuhan, China. This virus was previously named as 2019-nCoV and it is a positive, enveloped, single-strand RNA virus. It also shares a lot of similarities with two other coronaviruses, the MERS-CoV (Middle East Respiratory Syndrome) and SARS-CoV (Severe Acute Respiratory Syndrome). Outbreak of the COVID-19 started with the report of a first suspected case on December 8, 2019 in Wuhan. The first two months of the epidemic covered three significant holidays, including the New Year of 2020, the Chinese New Year’s Day with vacations from January 24 to February 2, 2020, and the Lantern Festival on February 8, 2020. During this period, one study by the Chinese Center for Disease Prevention and Control (CDC) and Hubei Provincial CDC with data collected by Wuhan CDC documented the details of the epidemic day by day from December 8, 2019 to January 21, 2020 [1]. Data in this study showed that detected and confirmed cases with COVID-19 infection declined from the peak of 44 on January 8 to only 2 on January 19, 2020, suggesting that the epidemic was likely under control.

China officially declared the epidemic as an outbreak on January 20 when obvious human-to-human transmissions were ascertained with reagent probes and primers distributed to local agencies on that day. Immediately following the declaration, massive actions were taken the next day to curb the epidemic at Wuhan, and soon spread to the whole country from central to local government, including all sectors from business to factories and to schools. On February 23, 2020, Wuhan City and other cities along with the main traffic lines around Wuhan were locked down. Rigorous efforts were devoted to 1) identify the infected and bring them to treatment in hospitals for infectious diseases, 2) locate and quarantine all those who had contact with the infected, 3) sterilize environmental pathogens, 4) promote mask use, and 5) release to the public of number of infected, suspected, under treatment and deaths on a daily basis.

On January 24, 2020, the New Year’s Eve and 25, the Chinese New Year’s Day, President Xi Jinping held a special meeting at the Central Chinese Government and decided to implement massive national efforts to curb the epidemic. An Anti- COVID-19 Group headed by Premier Li Keqiang was established to lead the massive national efforts. Vice Premier Sun Chunlan was sent to Hubei and Wuhan to directly lead the local efforts. A massive number of detection kits were made available to all locations to test all susceptible patients for final diagnosis. People in other cities and provinces who either traveled to or out of Wuhan were quarantined, with suspected patients being diagnosed and treated.

The sudden escalation of the control and the spread of the number of infected and deaths, however, ignited strong emotional responses of fear and panic among people in Wuhan. The negative emotional responses soon spread from Wuhan to other parts of China, and further to the world via almost all communication channels, particularly social media. The highly emotional responses of the public were fueled by (1) sudden increases in the number of detected new cases after the massive intervention measures to identify the infected; (2) massive growing needs for masks; (3) a large number of suspected patients waiting to confirm their diagnose; (4) a large number of diagnosed COVID-19 patients for treatment; and (5) a growing number of deaths, despite national efforts to improve therapy, including the decision to build two large hospitals within a period of days. The emotional responses, mostly stimulated by the daily release of data have created a big barrier for effective control of the epidemic as has been observed in other epidemics of similar nature [2, 3].

It is a paradox that during the early period of an epidemic, little is known or available about the new infections; while the need for such information is at the highest level. This is particularly true for the COVID-19. The occurrence of this epidemic may follow a nonlinear, chaotic and catastrophic process, rather similar to the epidemic of SARS that occurred in Hong Kong in 2003 [2], the Ebola epidemic in West Africa during 2013–16 [4, 5], the pandemic of 2009 H1N1 epidemic started [6–8] and the recent measles outbreaks in the United States (US) [9]. Similar to an eruption of a volcano or occurrence of an earthquake, no matter how closely it is monitored, how much research we have done, how much we know about it, no one knows for sure if and when the virus infection will become an outbreak. Therefore, there is no so-called rational responses, no standard-operating-procedure (SOP) to follow, no measures to take without negative consequences [2].

However, defining the COVID-19 as nonlinear and chaotic does not mean that we cannot do anything after we knew it was an outbreak, but simply waiting. On the contrary, defining it as nonlinear and chaotic will better inform us to make right decisions and to take appropriate actions. (1) During the early stage of an infection, which we cannot tell whether it will be growing into an outbreak, we must closely monitor it using limited data and to find the *early signs of change* and to predict if and when it will become an outbreak; (2) After it is declared as an outbreak, it is better to take actions as soon as possible since infectious diseases can be controlled even without knowledge of the biology [10]; and evaluate if the control measures work.

The ultimate goal of this study is to attempt to provide some solutions to this paradox by providing early messages to inform control measures, to be optimistic and not panic, to ask right questions, and to take right actions.

## Methods

### Daily detected and confirmed cases

Data for this study were daily cumulative cases with COVID-19 infection for the first two months (63 days) of the epidemic from December 8, 2019 to February 8, 2020. These data were derived from two sources: (1) Data for the first 44 days from December 8, 2019 to January 20, 2020 were derived from published studies that were determined scientifically [1]. Since no massive control measures were in place during this period, these data were used as the basis to predict the underlying epidemic, considering the overall epidemic. The best fitted model was used to predict the detectable cases and was used in assessing detection rate at different periods for different purposes.

Data for the remaining 19 days from January 21 to February 8, 2020 were taken from the daily official reports of the National Health Commission of the People’s Republic of China (http://www.nhc.gov.cn/xcs/yqfkdt/gzbd_index.shtml). These data were used together with the data from the first source to monitor the dynamic of COVID-19 on a daily basis to 1) assess whether the COVID-19 epidemic was nonlinear and chaotic, 2) evaluate the responsiveness of the epidemic to the massive measures against it, and 3) inform the future trend of the epidemic.

### Understanding of the detected cases on a daily basis

In theory, the true number of persons with COVID-19 infection can never be known no matter how we try to detect it. In practice, of all the infected cases in a day, there are some who have passed the latent period when the virus reaches a detectable level. These patients can then be detected if: a) detection services are available to them, b) all the potentially infected are accessible to the services and are tested, and c) the testing method is sensitive, valid and reliable. When reading the daily data, we must be aware that the detected and diagnosed cases in any day can be great, equal, or below the number of detectable. For example, a detectable person in day one can be postponed to next day when testing services become available. This will result in reduction in a detection rate < 100% in the day before the testing day and a detection rate > 100% in the testing day.

### Model daily change in the epidemic

We started our modeling analysis with data of cumulative number of diagnosed COVID-19 infections per day. Let *x*_*i*_ =diagnosed new cases at day *i*, *i* =(1, 2, …*t*), the cumulative number of diagnosed new cases *F*(*x*) can be mathematically described as below:

1 $$ F(x)={\int}_{i=1}^t{x}_i=\sum \limits_{i=1}^t{x}_i. $$

Results of *F*(*x*) provide information most useful for resource allocation to support the prevention and treatment; however *F*(*x*) is very insensitive to changes in the epidemic. To better monitor the epidemic, the first derivative of *F*(*x*) can be used:

2 $$ F^{\prime }(x)={\int}_{i=1}^{\left(t+1\right)}{x}_i-{\int}_{i=1}^t{x}_i=\sum \limits_{i=1}^{t+1}{x}_i-\sum \limits_{i=1}^t{x}_i $$

Information provided by the first derivative *F* ′ (*x*) will be more sensitive than *F*(*x*), thus can be used to gauge the epidemic. Practically, *F* ′ (*x*) is equivalent to the newly diagnosed cases every day. A further analysis indicates that *F* ′ (*x*), although measuring the transmission speed of the epidemic, provides no information about the acceleration of the epidemic, which will be more sensitive than *F* ′ (*x*). We thus used the second derivative *F*″(*x*):

3 $$ {F}^{{\prime\prime} }(x)={F}^{\prime}\left({x}_{\mathrm{i}+1}\right)-{F}^{\prime}\left({x}_i\right) $$

Mathematically, *F*′′(*x*) measures the acceleration of the epidemic or changes in new infections each day. Therefore, *F*^′′^(*x*) ≈ 0 is an early indication of neither acceleration nor deceleration of the epidemic; *F*^′′^(*x*) > 0 presents an early indication of acceleration of the epidemic; while *F*^′′^(*x*) < 0 represents an early indication of deceleration.

### Modeling the epidemic with assumption of no intervention

With a close population assumption and continuous spread of the virus, the number of detected cases can be described using an exponential model [10]. We thus estimated the potentially detectable new cases every day for the period by fitting the observed daily cumulative cases to an exponential curve:

4 $$ F\left(\overline{x}\right)=\left(\alpha \right){\mathit{\exp}}^{\beta (t)},\mathrm{t}=\left(12/8/2019,12/9/2019,\dots, 1/20/2020\right), $$

where, *α* =number of expected cases at the baseline and *β* = growth rate per day.

### Estimation of daily detection rate

To assess the completeness of the diagnosed new cases on a daily basis, we used Eq (4) first to obtain a time series of $F\left(\overline{x}\right)$ to represent the estimates of cumulative number of potentially detectable cases; we then used the first derivative $F^{\prime}\left(\overline{x}\right)$ to obtain another time series of observed new cases each day; finally, with the observed *F* ′ (*x*_*i*_) and model predicted $F^{\prime}\left(\overline{x}\right)$, we obtained the *detection rate P*_*i*_ for day *i* as:

5 $$ {P}_i=F^{\prime}\left({x}_i\right)/{F}^{\prime}\left({\overline{x}}_i\right),\mathrm{i}=\left(12/8/2019,12/9,2019\dots, 2/8/2020\right) $$

We used these estimated *P*_*i*_ in this study in several ways.

1. Before January 20, 2020 when the massive intervention was not in position, an estimated *P*_*i*_ > 1 was used as an indication of detecting more than expected cases, while an estimated *P*_*i*_ < 1 as an indication of detecting less than expected cases.
2. During the early period of massive intervention, an increase trend in *P*_*i*_ over time was used as evidence supporting the effectiveness of the massive intervention in detecting and quarantining more infected cases.
3. During the period 14 days (latent period) after the massive intervention, *P*_*i*_ < 1 was used as evidence indicating declines in new cases rather than under-detection; thus, it was used as a sign of early declines in the epidemic.

The modeling analysis was completed using spreadsheet. As a reference to assess the level of severity of the COVID-19 epidemic, the natural mortality rate of Wuhan population was obtained from the 2018 Statistical Report of Wuhan National Economy and Social Development.

## Results

### Cumulative number of detected and diagnosed cases

The COVID-19 epidemic was initiated in Wuhan, the Provincial Capital of Hubei Province with a total population of 14.2 million, including 5.1 million mobile population. The mortality rate was 5.5/1000 for Wuhan residents with most available data in 2018. Assuming all diagnosed cases in China were infected in Wuhan (an exaggerated scenarios for illustration purpose), the two-month incidence rate of COVID-19 was 2.6/1000 among Wuhan residents. Based on reported case mortality of 2.3%, the population-based mortality of COVID-19 was 0.6/1000, or 1/9th of the mortality of Wuhan residents.

**Fig. 1:**
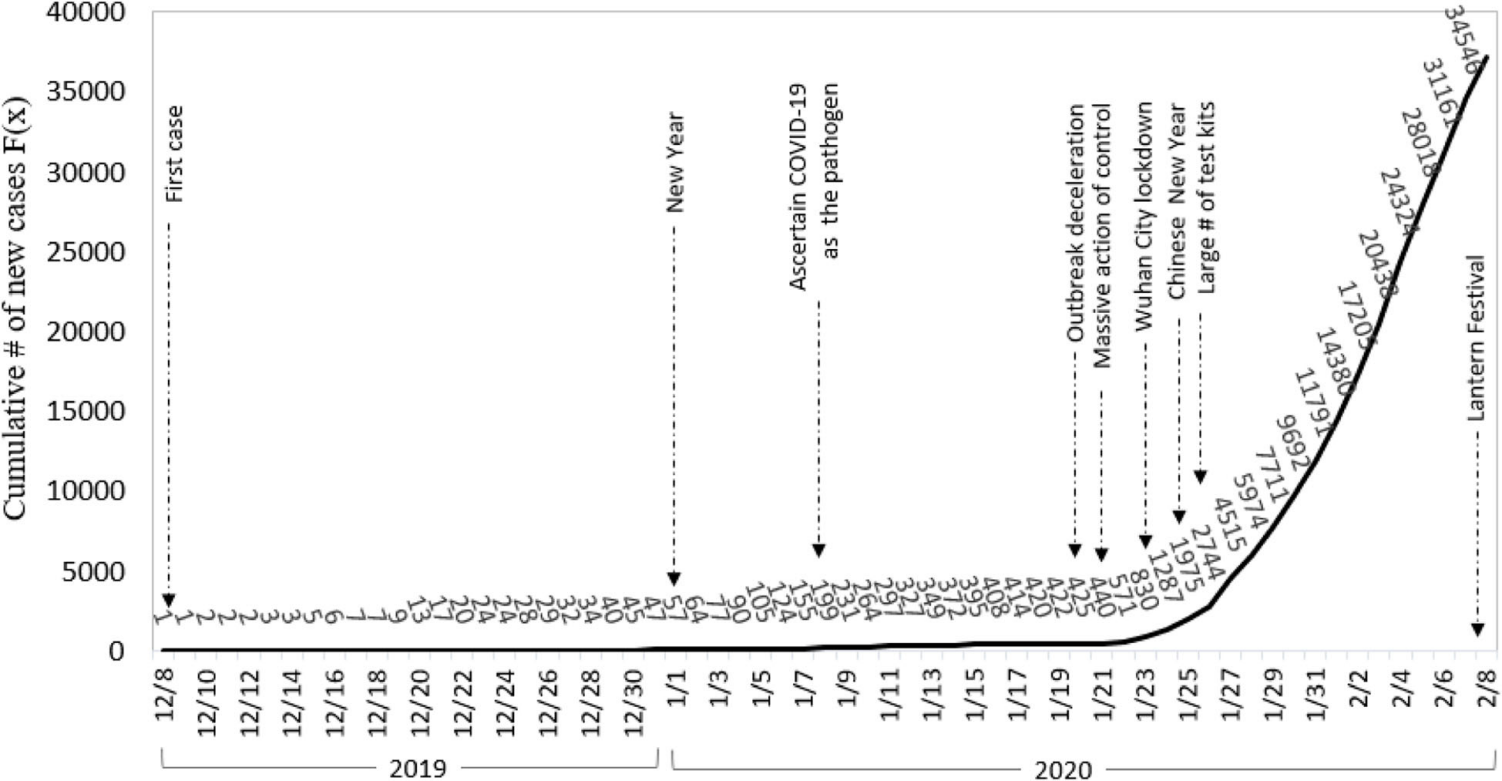
Cumulative number of diagnosed COVID-19(2019-nCoV) infection F(x) and key events before, during and after declaration of the outbreak in the first 2 months of the Epidemic in China

Figure 1 presents the cumulative diagnosed cases F(x) and major events during the study period from December 8, 2019 to February 8, 2020. During the period, a total of 37,198 cases were diagnosed and reported. The daily cases varied from 0 to 3886 with the median cases of 199 (January 8, 2020), and inter-quarter range (IQR) of 24 (December 23), and 830 (January 23, 2020).

### Dynamics of the epidemic and response to massive interventions

**Fig. 2:**
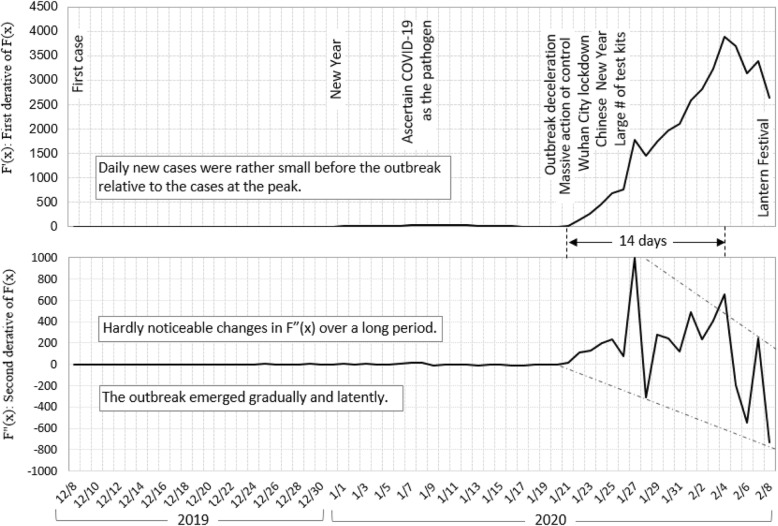
The first F′(x) and second derivative F″(x) of diagnosed COVID-19 (formally 2019-nCoV infection) F(x) before, during and after declaring the outbreak in first 2 months of the Epidemic

The dynamic changes based on the observed *F*(*x*) in Fig. 1 were presented in Fig. 2 using the first derivatives *F* ′ (*x*) (top panel of the figure) and the second derivative *F* ′  ′ (*x*) (bottom panel of the figure), respectively. Before the declaration of outbreak, information provided by the two dynamic measured was similar: not much variations were revealed relative to the changes after the outbreak. These findings suggest the nonlinear and chaotic character of the COVID-19 outbreak.

After declaring the outbreak on January 20, information revealed by *F* ′  ′ (*x*) differed much from *F* ′ (*x*). Based on information from *F* ′ (*x*), the newly diagnosed *F* ′ (*x*) cases increased progressively with some fluctuation, then peaked on February 4, 2020, and followed by a decline. The increases in the diagnosed cases could be either due to the natural growth of the epidemic in itself, or due to the interventions to detect the infected or both. Furthermore, *F* ′ (*x*) provided no sign of epidemic decline until February 4, 2020. In other words, we have to wait for at least 14 days after the massive anti-COVID-19 epidemic without using information derived from *F* ′  ′ (*x*).

Quite different from *F* ′ (*x*), *F* ′′ (*x*) removed the time trend of *F* ′ (*x*) to show the acceleration/deceleration of diagnosed COVID-19. Consequently, *F* ′  ′ (*x*) was much more sensitive than *F* ′ (*x*) to gauge the intrinsic dynamics of the epidemic in response to the massive anti-COVID-19 action. Since January 21, 2020 after the massive anti-COVID actions, the *F* ′  ′ (*x*) suddenly became very active, as indicated by the alternative accelerations and decelerations. *F* ′  ′ (*x*) reached the peak on January 27 after the distribution of large number of test kits on January 26, which was an action based on the decision at the central government level in a meeting held by Chinese President Xi Jinping on January 24 and 25, the Chinese New Year’s Eve and New Year’s Day.

In addition, the estimated *F* ′′ (*x*) captured three significant decelerations on January 28, February 5 and 6 (two days in a row), and 8, 2020 respectively; corresponding to the intensified massive actions in locating and treating the infected, locking down more communities, plus mask use and massive pathogen sterilization in neighborhood environment. In addition to informing whether the epidemic was responsive to the massive interventions, information from *F* ′′ (*x*) signaled an overall downturn of the epidemic since the beginning of the massive anti-COVID-19 action on January 21, 2020. This was further pronounced by the band region between the two dotted lines in Fig. 2. Despite zigzags, an overall downward trend in *F* ′′ (*x*) was clearly revealed by the downward and progressively narrowing down band region. This trend strongly indicates that the epidemic could be brought under control soon with the current interventions in place.

### Exponential growth and detection rate

The observed *F*(*x*) fit the exponential model of Eq. 4 well with *R*^*2*^ = 0.9778. The estimated *α* =1.1070, representing the first person who was infected and ignited the epidemic. The estimated *β* =0.1716, representing the growth rate. Using this estimated growth rate, it takes only 4 days for the diagnosed COVID-19 to double.

**Fig. 3:**
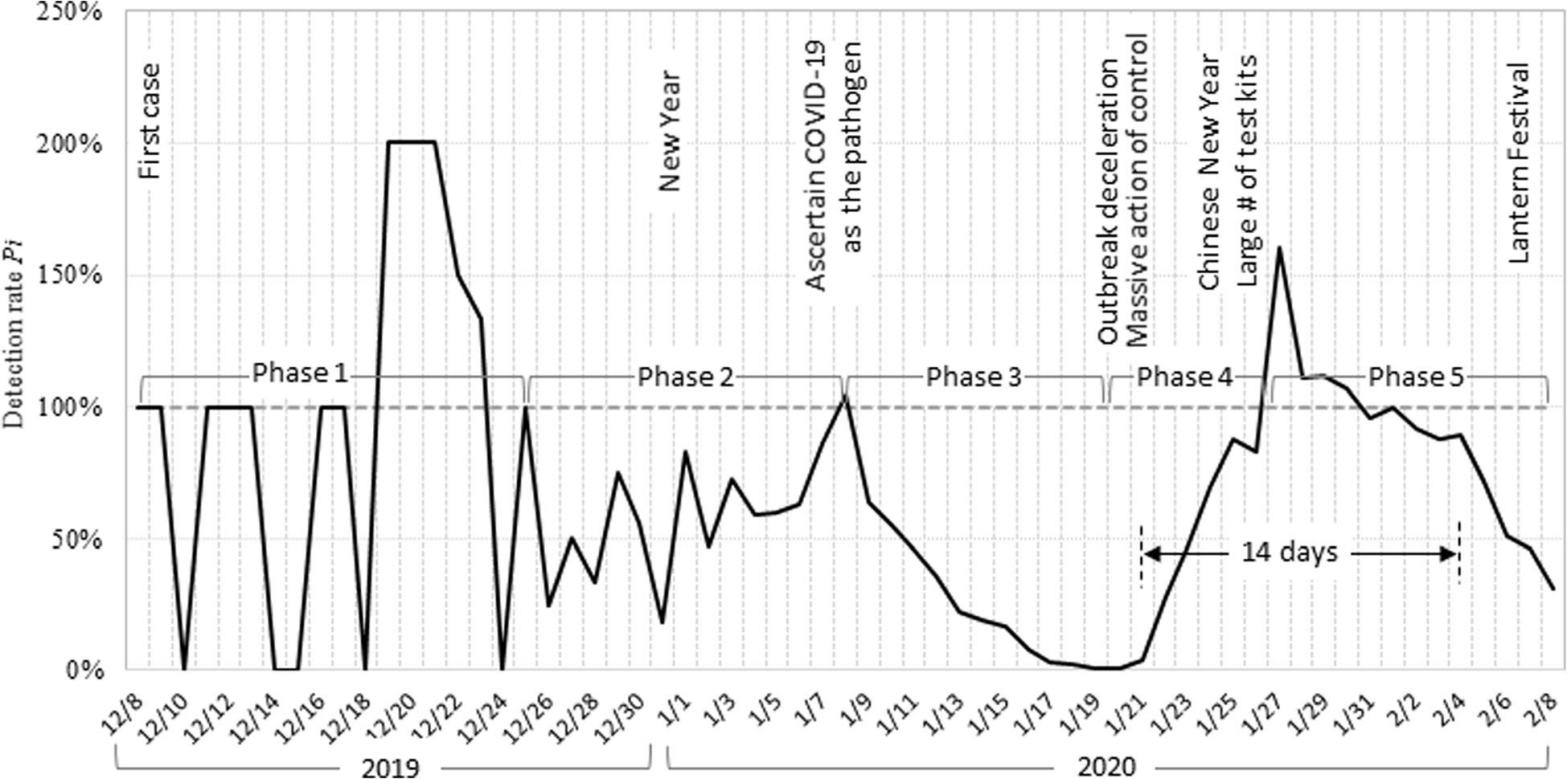
Estimated daily detection rate *Pi* of COVID-19 (2019-nCoV) infection before, during and after declaration of the outbreak, the first 2 months of the Epidemic in China

Figure 3 presents the daily detection rates, estimated with the fitted exponential model from day one of the epidemic to the last day of the study period. Based on findings in this figure and data from Figs. 1 and 2, we divided the COVID-19 epidemic during the first two months of the epidemic into five phases.

Phase 1 was from December 8 to 25, 2019. During this period, the detection rate *P*_*i*_ was high overall, with fluctuations around and above 100%. This was corresponding to the early period after the first suspected case was identified and diagnosed.

Phase 2 was from December 26, 2019 to January 8, 2020, covering the New Year’s Day. The detection rate *P*_*i*_ fluctuated at around 50% with the lowest of 17% on December 31, 2019 and the highest of 108% on January 8.

Phase 3 was from January 8 to 20, 2020, and it was featured with a progressive decline in the estimated *P*_*i*_ from 105% on January 8, 2020 to 1% on January 20, 2020. This progressive declining period was the time for the Chinese to prepare for the traditional Chinese New Year’s with the longest and highest level of celebration. Unfortunately, the COVID-19 as an outbreak was silently stepping in during this period.

Phase 4 was from January 20 to 27, 2020 with the estimated *P*_*i*_ increased from 1% on January 20, 2020 to surpass 100%, and reached the peak of 170% on January 27, 2020. This period was corresponding to the initiation and progressive intensifying of the massive intervention organized and coordinated by the Central Government of China.

Phase 5 started from January 27, 2020 to the end of the study period, corresponding to the sustained massive national efforts, plus frequent emphases. Different from the previous four phases, reductions in the estimated *P*_*i*_ during this phase were *not an indication of under-detection* but an indication of *declines in the epidemic* reflected by the detected and confirmed cases of COVID-19. This is because the model predicted *P*_*i*_ did not consider any interventions but natural growth of the epidemic.

Based on Figs. 2 and 3 (Phase 4 and 5), three pieces of information can be derived: (1) The epidemic was highly sensitive to external interventions, supporting the nonlinear and chaotic characters revealed by the long latent period in the first three phases; (2) the massive national efforts were highly effective in detecting the detectable COVID-19; (3) signal for the COVID-19 in China to decline appeared on January 21 in 2020, 14 days before the start of eventual declines on February 4, as indicated by *F* ′  ′ (*x*) and *F* ′ (*x*) in Fig. 2 and *P*_*i*_ in Fig. 3.

## Disscussion

In this study, we used a novel approach to distill information from the cumulative number of diagnosed cases of COVID-19 infection. Among various types of surveillance data, this data often reported the earliest and on a continuous basis with high completeness and are most widely available. In addition, patients with a diagnosed infection are those with high likelihoods to spread the virus to others. Findings from this study provided useful information in a real time manner to monitor, evaluate and forecast the COVID-19 epidemic in China. The methods used in this study although somewhat mathematical, are easy to follow while information extracted from the commonly used data with the methods are highly useful and more sensitive than the daily new and cumulative cases.

### Nonlinear and chaotic nature of the COVID-19 outbreak

Although an analytical demonstration of the COVID-19 outbreak as nonlinear, chaotic and catastrophic requires more time to wait till the epidemic ends, evidence in the first 2 months suggests that the COVID-19 outbreak in China is nonlinear and chaotic. The epidemic emerged suddenly after a long latent period without dramatic changes as revealed from the cumulative cases, and their first and second derivatives. The high responsiveness of the epidemic to interventions adds additional evidence supporting the chaotic and catastrophic nature, and demonstrating the selection of a good timing to start intervention. Many of these characters are similar to those observed in the 2003 SARS epidemic started in Hong Kong [2], the 2013–16 Ebola spread in the West Africa [4, 5], the 2009 pandemic of H1N1 started in the US [6–8], and the measles outbreaks over 80 cities in the US recently [9]. Even the seasonal common flu has been proved to have a nonlinear component [11, 12].

The significance of nonlinear and chaotic nature of COVID-19 means that no methods are available to predict exactly at what point in time the epidemic will emerge as an outbreak, just like volcanoes and earthquakes. Therefore, practically there is no so-called a *best time* or missed the best time to take actions. There will also no so-called *rational analysis and rational responses*. There is no *silver bullet* to use, no *standard-operating-procedure* (SOP) to follow, and *no measures without negative consequences* to control the epidemic [2]. For example, it took more than 6 months for both the US and the WHO to determine the 2009 H1N1 pandemic as an outbreak [13, 14]. Therefore, knowing the nonlinear and chaotic nature of an epidemic outbreak, like COVID-19, for all stockholders will be essential to the mobilization of resources, working together, taking all actions possible to control the epidemic, and minimizing the negative consequences.

Specifically, what we can do to deal with an outbreak like COVID-19 would be to (1) collect information as early as possible, (2) monitor the epidemic as close as possible just like we do for an earthquake and make preparations for a hurricane and (3) communicate with the society and use confirmed data appropriately reframed not causing or exacerbating fear and panic in the public, stress and distress among medical and public health professionals, as well as administrators to make right decisions and take the right strategies at the right time in the right places for the right people.

Knowing the nonlinear and chaotic nature is also essential for taking actions to control the outbreak of an epidemic like the COVID-19 infection. As soon as an outbreak is confirmed, the follow measures should be in position immediately 1) closely and carefully monitor the epidemic; 2) take evidence-based interventions to control the epidemic, 3) actively assess responses of the epidemic to the interventions; 4) allow errors in the intervention, particularly during the early period of the epidemic, 5) always prepare for alternatives.

Another confusion is, when an epidemic starts, everyone asks what it is? How does it happen? How should I do to avoid infection? Is there any effective treatment? Answering these questions takes time, but there is no need to wait till all these questions are resolved before taking actions. We can take actions to prevent COVID-19 immediately while waiting for answers to these questions. This is because we have the evidence-based strategy for control and prevention of any infectious disease without complete understanding of an infection. That is so-called Tri-Component Strategy: locating and controlling the sources of infection, identifying and blocking the transmission paths, and protecting those who are susceptible [10].

This was just what China has done, is doing, and will continue to do this time. Typical examples of control and prevention measures include locking down of cities, communities, and villages with potential of large scale transmission, massive environment sterilization, promotion of mask use, efforts to locate, isolate and treat the infected. More importantly, most of these actions are initiated, mobilized, coordinated and supported by the government from central to local, and enhanced by volunteers and international support.

### Highly effective of the national effort

Another important piece of findings is that we detected the effect of the national efforts taken by China from the beginning when they were in position till the end of this study. For example, from the second derivative, we observed increases in the infected through the action on January 22, 2020, the next day after the massive intervention started on January 21, 2020. This result was also picked up by the exponential modeling. From day one on January 21, 2020 when the massive intervention measures activated to February 4, 2020 is the latent period of COVID-19 infection. The second derivative precisely recorded the change in newly diagnosed cases in response to the massive measures, reflected as the rapid increase in detection rate, consistent with the result from the exponential modeling analysis.

The detected responsiveness of the epidemic to the intervention provided data to predict the occurrence of deceleration of the epidemic on February 4, 2020 if the same measures persist, which was exactly what we observed from the second derivative. Based on the findings from our analysis, the COVID-19 in China may end up soon. Despite a delay of 43 days from the first confirmed cases on December 8, 2019 to January 20, 2020, the COVID-19 epidemic was highly responsive to massive interventions, supporting the effectiveness of these interventions. It is our prediction that the outbreak of the COVID-19 infection will be brought under control by the end of February 2020, given the effective control measures known to everyone, increases in immune level in the total population due to latent infections, and most widely spread of knowledge and skills for infectious disease control and prevention among the 1.4 billion people in China.

### Effective methods for surveillance

There are a number of advantages of methods we developed and used in this study. First, framing the diagnosed cases as the cumulative, the first and the second derivative constructs a system to gauge the epidemic, with the cumulative cases showing the overall level of the epidemic, the first derivative to reflect the change of the epidemic, and the second derivative to monitor the speed of change. By inclusion of the mortality rate as a reference, results from our approach will be (1) comprehensive to inform the public to be prepared, not scared and not to blame others; (2) useful for administrators to make decisions; (3) valuable for medical and health professionals to take actions.

Second, we conceptually separated (1) the true number of infections, which will never be practically detected, from (2) the infections that are practically detectable if services are available and accessible and detection technologies are sensitive and reliable, and (3) the actually detected cases of infections. This classification greatly improved our understanding of the observed data as well as findings from the two derivatives, and aided us in assessing the responsiveness to the massive interventions, and predicting of the epidemic over time. The clarification also enhanced our analytical approach by adding an exponential model to evaluate the detection rate and to bring more data assessing the responsiveness of the epidemic to the massive interventions. We highly recommend the inclusion of the methods as a part of routine surveillance in disease control and prevention institutions.

### Limitations and future plan

There are limitations. First, this study covered only the first 2 months of the epidemic. We will continue to evaluate the utility of this method as we follow the development of the epidemic. Second, the methods used in this study was based on a close population. This hypothesis may not be true because of a large number of people with potential history of exposure in China traveled to other countries. Up to February 8, 2020, the total cases diagnosed were 37,552 worldwide (Worldometer on Coronavirus) with 37,198 in China, which accounted for 99.1% of the total number of the world. Therefore, the impact of close-population assumption would be rather limited. Third, there was a lack of individual patient-level data for detailed analysis. Fourth, our model can be further improved with other data, such as cases by severity, number of the suspected, number of those who received treatments and treatment results. We will follow the epidemic closely and prepare for further research on the topic when more data become available.

Despite the limitations, this study provided new data to encourage those who are infected to better fight against the infections; to inform and encourage the general public, the medical and health professionals and the government to continue their current measures and to think of more measures that are innovative and effective to end the COVID-19 epidemic. One of the greatest motivations for this study is to attempt to provide right information at the population level in a real manner to complement the data from micro-organism centered and laboratory-based biological, molecular, pharmacological and clinical information in both the academic and the mass media that often scare rather than encourage people, even health professionals. Of the diagnosed COVID-19 cases, less than 20% are severe. Findings from our study indicated that there is no need to be panic from a public health population perspective. Although the total cases COVID-19 reached to big numbers, but the 2-month incidence rate was about a half of the natural death rate for Wuhan residents.
